# Pyrenochaeta unguis-hominis-associated fungal keratitis: A rare case report with in vivo confocal microscopy findings

**DOI:** 10.1016/j.ajoc.2025.102373

**Published:** 2025-06-28

**Authors:** Andreas Guttmann, Paul Wintersteller, Nora Woltsche, Astrid Heidinger, Nika Medic Ajdnik, Karin Pekovits, Ingrid Boldin, Haleh Aminfar, Jutta Horwath-Winter

**Affiliations:** Department of Ophthalmology, Medical University Graz, Auenbruggerplatz 4, 8036, Graz, Austria

**Keywords:** In vivo confocal microscopy, Fungal keratitis, Pyrenochaeta unguis-Hominis, Neocucurbitaria unguis-hominis

## Abstract

**Purpose:**

Pyrenochaeta unguis-hominis, also known as Neocucurbitaria unguis-hominis, is a rare fungal pathogen typically isolated from skin and nail infections. Recently, it has been identified as a cause of fungal keratitis, particularly among contact lens wearers. This case report documents the occurrence of Pyrenochaeta unguis-hominis keratitis in Austria and the visualization of changes in the corneal stroma using in vivo confocal microscopy (IVCM).

**Observations:**

A 48-year-old female patient presented with severe photophobia and acute pain in her left eye, following extended wear of soft contact lenses. Initial examination revealed a central corneal infiltrate. IVCM was performed prior to corneal scraping, which was then sent for direct staining, culture, and next-generation sequencing (NGS) and identified Pyrenochaeta unguis-hominis and Streptococcus oralis. Treatment included hourly topical voriconazole 2 %, natamycin 5 % and vancomycin 2.5 %, with additional epithelial debridement to enhance drug penetration. IVCM imaging allowed for real-time visualization and tracking of structures with the appearance of fungal hyphae, guiding the treatment course. Over several months, IVCM demonstrated a reduction in these structures, and the patient's condition stabilized, resulting in improved corneal clarity and Best Corrected Distance Visual Acuity from 0.8 to 0.9 (Snellen decimal scale).

**Conclusions and importance:**

This case contributes to the limited clinical literature on Pyrenochaeta unguis-hominis-associated keratitis and includes IVCM imaging of a cornea with this rare infection. While IVCM provided early, non-invasive visualization of stromal changes, definitive diagnosis was achieved through molecular testing. A conservative treatment regimen with topical antifungals and epithelial debridement was effective, emphasizing the importance of rapid diagnostics and targeted therapy in managing rare corneal infections.

## 1 Introduction

Pyrenochaeta unguis-hominis, also known by its reclassified name Neocucurbitaria unguis-hominis, is a rare fungal pathogen typically isolated from skin and nail infections, such as onychomycosis.^1^, ^2^, ^3^, ^4^, ^5^ First described in a case report from Spain in 2009, Pyrenochaeta unguis-hominis has now been implicated only in a few keratitis cases worldwide.^5^

Infectious keratitis is a vision-threatening ocular condition, with delayed or inadequate treatment often leading to severe complications.^6^ While fungal keratitis has historically been more prevalent in tropical and subtropical regions, its incidence is increasing in Europe and the United States.^6^, ^7^, ^8^, ^9^ In developing countries, fungal keratitis remains the leading cause of corneal blindness.^7^^,^^9^ With shifting environmental factors, such as climate change, and an increase in risk factors like contact lens wear, it is plausible that previously rare or exotic fungal species may now cause fungal keratitis in Europe.^7^^,^^8^^,^^10^, ^11^, ^12^, ^13^, ^14^

Data from the German Fungal Keratitis Registry show that only about 20 % of cases are correctly diagnosed at the initial ophthalmic consultation, with bacterial keratitis, herpetic keratitis, or acanthamoeba keratitis often considered as differential diagnoses.^6^ The correct diagnosis of fungal keratitis is made between 10 and 32 days after symptom onset, significantly delaying the initiation of appropriate antifungal therapy.^6^^,^^15^, ^16^, ^17^, ^18^, ^19^ This delay underscores the diagnostic challenges even for corneal specialists, as fungal keratitis can be difficult to distinguish from other infectious causes.^7^

While culturing pathogens from corneal scrapings remains the gold standard for diagnosing fungal keratitis, it is time-consuming and often delays treatment.^19^^,^^20^ In vivo confocal microscopy (IVCM) offers a significant advantage by enabling rapid, non-invasive visualization of fungal elements within the corneal stroma in real-time.^20^ This technology provides with a sensitivity of 71–98 % in advanced observer early diagnostic insights, facilitating the prompt initiation of targeted antifungal therapy and improving patient outcomes.^6^^,^^21^

After conducting a literature review on Nov. 8, 2024 utilizing PubMed, Google Scholar, and Embase using the keywords Neocucurbitaria unguis-hominis, keratitis, Pyrenochaeta unguis-hominis, we did not find any prior reports of IVCM imaging of this pathogen. This case represents a documentation of Pyrenochaeta unguis hominis fungal keratitis in Austria, and IVCM images from a female patient with a history of contact lens wear with this rare infection.

## 2 Case presentation

A 48-year-old female patient presented to the Department of Ophthalmology at the Medical University of Graz with severe photophobia and acute pain in her left eye. She had a 30-year history of wearing soft contact lenses, with recent continuous use of “Day&Night” lenses for over 30 days. Initial slit-lamp examination revealed a central, fluffy corneal infiltrate measuring 1 × 1 mm, and her Best Corrected Distance Visual Acuity (BCDVA) was 0.8 (Snellen decimal scale). The patient had no prior history of eye infections, trauma, or any systemic disease, but her prolonged contact lens use and her gender placed her at an elevated risk for microbial keratitis.^6^

**Fig. 1:**
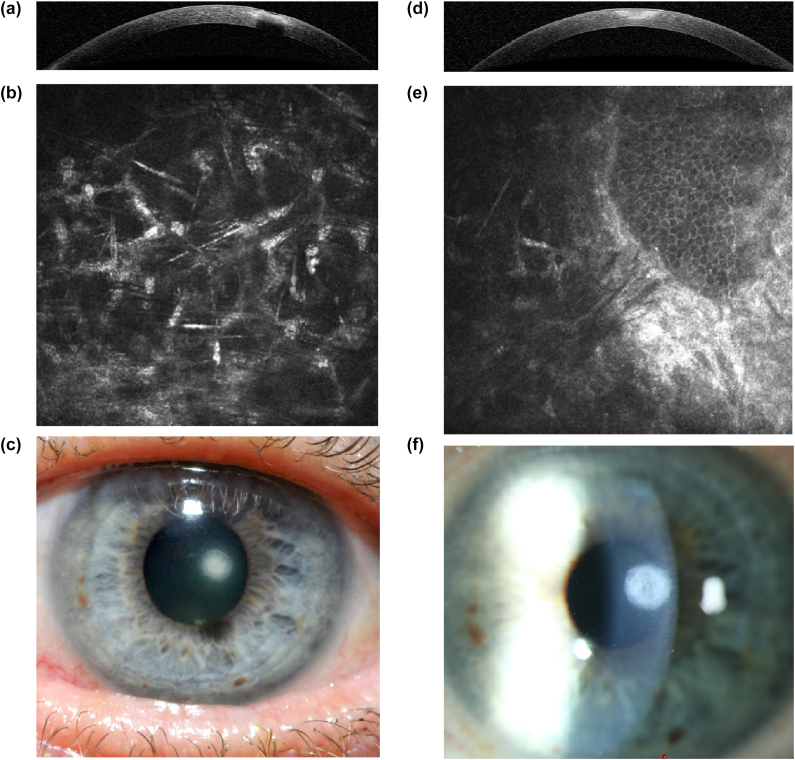
Imaging of *Pyrenochaeta unguis-hominis*-associated keratitis at presentation and one year follow-up.

Based on the clinical appearance, a corneal scraping was performed for microbiological analysis. Samples were subjected to direct staining (Gram, Periodic Acid-Schiff (PAS), Lactophenol Cotton Blue (LPCB)), culture, and next-generation sequencing (NGS). The initial Gram stain indicated the presence of Gram-positive cocci. However, PAS staining revealed fungal hyphae, raising the suspicion of fungal keratitis. Additionally, IVCM provided early evidence in the assessment by detecting structures suggestive of fungal elements deep within the corneal stroma and played an important role in follow-up examinations by allowing visualization of changes during the course of therapy. According to the classification by Bakken et al. (2022), these findings align with Category 2, supporting a diagnosis of possible fungal keratitis (Fig. 1A–C).^22^ Treatment was initiated with hourly voriconazole 2 % as well as gentamycin and ofloxacin eye drops every hour.

Comparison of images of fungal keratitis at initial presentation (A-C) and the scarred healing one year later (D-F) after consistent treatment with antibiotics, antifungals, and steroids using AS-OCT (A,D), in vivo confocal microscopy (B,E), and slit lamp photography (C,F).

After a few days, slight improvement was observed, and the therapy was reduced. Shortly thereafter, NGS confirmed Pyrenochaeta unguis-hominis, which was performed at the Diagnostic and Research Center of Molecular BioMedicine of the Medical University of Graz following amplification with ITS1 and ITS2 primers. Additionally, microbiology identified Streptococcus oralis, which was sensitive to vancomycin. An epithelial debridement was performed, and the therapy was adjusted to preservative-free ofloxacin eyedrops 4 times daily and natamycin 5 %, voriconazole 2 %, and vancomycin 2.5 % eyedrops every hour.

Within days, the corneal infiltrate showed sharper borders. A subsequent epithelial debridement was performed, and ofloxacin was discontinued while hourly voriconazole and natamycin and six times vancomycin were continued. Over the following days, the infiltrate became more defined, leading to a reduction in the frequency of voriconazole and natamycin to six times daily and vancomycin 4 times daily, with the addition of a steroid ointment at night.

Stability was achieved, and steroid drops were added to the morning regimen. As the corneal epithelium began to close, another epithelial debridement was performed to enhance medication penetration. A few weeks later, the structures with the appearance of fungal hyphae were still visible via IVCM up to a depth of 320 μm, necessitating further epithelial debridement.

Following clinical improvement, the frequency of voriconazole and natamycin was gradually reduced. The patient's condition remained stable, and the epithelium eventually closed completely, with the scar showing mostly sharp, defined borders. The patient was monitored closely over the next several months. Regular IVCM imaging demonstrated a marked reduction in the number and size of the structures with the appearance of fungal hyphae, correlating with clinical improvement. The regimen was further adjusted by tapering the antifungal agents while continuing preservative-free lubricating drops and steroid drops. At this time, vancomycin eye drops were discontinued.

After 11 months, slit-lamp examination revealed significant improvement in corneal clarity, with scar formation and no signs of active infection (Fig. 1D–F). The BCDVA improved to 0.9 (Snellen decimal scale).

## 3 Discussion

This case of Pyrenochaeta unguis-hominis highlights key aspects in the management of rare fungal infections. The diagnosis and therapy regimen of fungal keratitis can be challenging due to the rarity of some pathogens and the difficulty in differentiating fungal from bacterial infections in the early stages of the disease.^11^ In this case, IVCM and direct staining played a crucial role in the early identification of fungal elements, enabling timely initiation of appropriate therapy.

The therapeutic approach in this case differed from the previously documented cases in Spain and Belgium, where systemic antifungals and surgical interventions, such as amniotic membrane transplantation and penetrating keratoplasty, were required due to severe corneal damage and persistent epithelial defects.^10^^,^^14^

In contrast, the present case was successfully managed with a conservative, non-surgical treatment approach, aided by the absence of inflammatory cells or hypopyon in the anterior chamber. The patient in this case responded well to topical treatment with voriconazole 2 % and natamycin 5 %, supported by mechanical debridement, without the need for surgery or oral antifungals. These two antifungal agents are commonly used for fungal keratitis. Natamycin is the first-line treatment for filamentous fungi. Studies, such as the Mycotic Ulcer Treatment Trials (MUTT I and II) demonstrated that natamycin was superior to voriconazole, particularly in Fusarium ulcers, while for non-Fusarium cases, voriconazole was not significantly different from natamycin.^23^, ^24^, ^25^, ^26^

This case, which notably did not develop significant anterior chamber inflammation or hypopyon, was successfully treated with intensive topical therapy only, without the need for surgical intervention or adjunctive oral antifungal therapy. We speculate that other cases of keratitis caused by this organism may also respond to topical therapy if treatment is started early enough.^6^

Another important aspect of this case was the regular debridement of the corneal epithelium early in the treatment process. Mechanical debridement of the epithelium has been shown to enhance the penetration of topical antifungals into the deeper layers of the cornea, improving drug efficacy.^27^ By removing the infected and necrotic tissue, this technique can reduce the fungal load and allow better access for topical medications, contributing to the overall successful outcome. However, Prajna et al. (2010) conducted a multicenter, randomized, double-masked clinical trial comparing epithelial debridement versus no debridement and found no statistically significant difference in visual outcomes between the two groups.^24^^,^^27^The use of IVCM in our case also allowed a precise monitoring of the corneal healing process, ensuring that the antifungal therapy was effective without the need for additional invasive diagnostic procedures.

This underscores the effectiveness of a conservative approach when early diagnosis and limited corneal involvement are present. As interpretation of IVCM images remains subjective, clinical correlation and confirmation through microbiological testing are essential. Further studies are needed to establish the diagnostic reliability of IVCM in rare forms of fungal keratitis.

## 4 Conclusion

This case represents a documented instance of Pyrenochaeta unguis-hominis keratitis in Austria and IVCM images of a cornea with this rare infection. It contributes valuable information to the limited body of knowledge on Pyrenochaeta unguis-hominis keratitis and illustrates the role of IVCM as an adjunctive tool rather than a standalone diagnostic method. While IVCM aids in early assessment and therapy monitoring, its findings should always be confirmed through microbiological and molecular diagnostics. The successful outcome using a topical regimen of voriconazole 2 % and natamycin 5 %, supported by mechanical debridement, indicates that aggressive surgical interventions may be avoidable when early and accurate diagnosis is achieved. While corneal scarring was an unavoidable consequence, the patient's visual acuity was largely preserved, highlighting the importance of rapid diagnosis and treatment in preventing irreversible damage. Future research will be essential to determine standardized treatment protocols, particularly for rare fungal pathogens like Pyrenochaeta unguis-hominis.

## Claims of priority statement

After conducting a literature review on Nov. 8, 2024 utilizing PubMed, Google Scholar and Embase using the keywords (Neocucurbitaria unguis-hominis, keratitis, Pyrenochaeta unguis-hominis), we did not find any prior reports of in vivo confocal Microscopy imaging of this pathogen.

## CRediT authorship contribution statement

**Andreas Guttmann:** Writing – original draft, Methodology, Investigation, Data curation, Conceptualization. **Paul Wintersteller:** Writing – review & editing, Project administration, Investigation. **Nora Woltsche:** Writing – review & editing, Investigation. **Astrid Heidinger:** Writing – review & editing, Investigation. **Nika Medic Ajdnik:** Writing – review & editing, Investigation. **Karin Pekovits:** Writing – review & editing, Investigation. **Ingrid Boldin:** Writing – review & editing, Investigation. **Haleh Aminfar:** Writing – review & editing, Investigation. **Jutta Horwath-Winter:** Writing – review & editing, Supervision, Investigation, Conceptualization.

## Patient consent

Consent to publish this case report has been obtained from the patient in writing.

## Authorship

All authors attest that they meet the current ICMJE criteria for Authorship.

## Funding

No funding or grant support

## Declaration of competing interest

The authors declare that they have no known competing financial interests or personal relationships that could have appeared to influence the work reported in this paper.

## Abbreviations

AS-OCT: Anterior Segment-Optical Coherence Tomography
BCDVA: Best Corrected Distance Visual
DED: Dry Eye Disease
IVCM: In Vivo Confocal Microscopy
LPCB: Lactophenol Cotton Blue
MUTT: Mycotic Ulcer Treatment Trials
NGS: Next-Generation Sequencing
PAS: Periodic Acid-Schiff
